# Let There Be Light—Digital Eye Strain (DES) in Children as a Shadow Pandemic in the Era of COVID-19: A Mini Review

**DOI:** 10.3389/fpubh.2022.945082

**Published:** 2022-08-11

**Authors:** Sudip Bhattacharya, Petra Heidler, Sheikh Mohd Saleem, Roy Rillera Marzo

**Affiliations:** ^1^Department of Community and Family Medicine, All India Institute of Medical Sciences, Madurai, India; ^2^Department for Economy and Health, University for Continuing Education Krems, Danube University Krems, Krems an der Donau, Austria; ^3^Department of Health Sciences, St. Pölten University of Applied Sciences, St. Pölten, Austria; ^4^Independent Public Health Researcher, Srinagar, India; ^5^Department of Community Medicine, International Medical School, Management and Science University, Shah Alam, Malaysia; ^6^Global Public Health, Jeffrey Cheah School of Medicine and Health Sciences, Monash University Malaysia, Bandar Sunway, Malaysia

**Keywords:** computer vision syndrome, digital eye strain, digital eye syndrome, COVID-19, public health, pandemic

## Abstract

**Introduction:**

Digital eye strain, which is often ignored by the public, has emerged as a “Shadow Pandemic” in the era of the COVID-19 pandemic.

**Aim:**

The current paper is aimed at discussing the ill effect of digital screens on eyes in the wake of the COVID-19 pandemic.

**Methodology:**

A literature search was done using “PubMed,” “Google scholar”, and “Scopus” using key terms like “Digital Eye Strain,” “Eyestrain,” or “Computer Vision Syndrome.” Relevant articles were identified and included to support the argument for this narrative review.

**Results:**

Studies conducted in the UK reported that 68% of children extensively use computers, while 54% undertake online activities after the age of 3. Similar studies estimated 4 h and 45 min per day of screen exposure time among adults in the UK. Indian studies reveal that the prevalence of DES is 69% in adults and 50% in children respectively. Indian ophthalmologists found that computer-using and specialized ophthalmologists were more informed of symptoms and diagnostic signs but were misinformed about treatment modalities. The use of social media and multitasking is particularly prominent among younger adults, with 87% of individuals aged 20–29 years reporting the use of two or more digital devices simultaneously. It has been observed that the use of computer glasses corrects refractive errors and helps in the reduction of symptoms, while precision spectral filters help in reducing symptoms of micro-fluctuation of accommodation.

**Conclusion:**

We concluded that DES is emerging globally as a “Shadow Pandemic” and it is high time to respond. Community ophthalmologists, public health authorities, and educational sectors especially should be involved to prevent this.

## Introduction

The nationwide lockdown was extended and completed Phase 4 on May 31, 2020, to effectively limit the COVID-19 pandemic and flatten the curve in India as well as many other countries throughout the world. Throughout this lockdown, it was discovered that pupils' education was deteriorating, and it was necessary to consider how the specified curriculum might be fulfilled. The University Grants Commission (UGC) in India established two committees to oversee examinations and the academic calendar, as well as to encourage online learning. In addition, the UGC chairman advocated social distance, web-based learning, and e-education to prevent the spread of the ongoing COVID-19 pandemic.

With the outbreak of the COVID-19 pandemic, not only current University students, but also pupils from primary and secondary schools, were encouraged to participate in e-learning to complete the required curriculum on time. The recommendations published by the appropriate authorities were insufficient to instruct instructors and students on how and when to use e-learning methods. Without any limitations, our children are increasingly spending most of their time (almost 8 h each day) in front of computer or smart phone displays. E-learning approaches can have both positive and negative effects on our children's vision. In addition, students used online platforms for entertainment, communication, and information purposes during this pandemic. Due to the surge in COVID-19 cases, adults were encouraged to continue their work from home, and they were exposed to screens for a long duration. As result of home confinement, they used online platforms for entertainment, communication, and information.

Computer displays and smart phone screens generate blue light with wavelengths ranging from 380 to 500 nm, which can be hazardous to health. These high-energy waves can reach the eyes, causing everything from irritation to retinal damage. Dry eyes, impaired vision, headaches, near-sightedness, and eye fatigue are among the symptoms that can be induced by the dazzling effect of blue light. Digital eye strain (DES) or computer vision syndrome is the collective term for this (1).

A “collection of eye and vision-related issues that occur from extended computer, tablet, e-reader, and mobile phone usage,” according to DES, is an increasing public health hazard. When using digital screens for long periods of time, people may have minor to severe eye irritation and vision problems. The most prevalent symptoms of DES, according to the American Optometric Association, are eyestrain, headaches, impaired vision, dry eyes, and neck and shoulder pain (1).

The amount of time spent looking at a digital screen is directly related to eye pain. Many millions of people of all ages are at risk of DES due to the tremendous surge in digital gadget usage in recent years. While the symptoms are typically temporary, the illness can cause severe and regular pain for sufferers, as well as having major financial implications. Long-term exposure to blue light emitted by electronic gadgets, according to experts, can have serious consequences. Long-term exposure can cause photochemical damage to the eyes, which can lead to retinal cell destruction and make a person prone to age-related macular degeneration. Children are the most vulnerable age group.

With this goal in mind, we've put together this paper to talk about how long-term e-learning causes DES, how to correct accountability issues, and how to solve the problem.

## Methodology

In the aftermath of COVID-19, the current study attempted to address the impact of digital displays on the eyes. The key phrases DES, Eye strain, and computer vision syndrome were searched for in the “*PubMed,” “Google Scholar,”* and “*Scopus”* databases. To complement the narrative review, all relevant articles were included in this publication.

## Results

Children typically have uncorrected vision difficulties such as farsightedness and astigmatism, insufficient eye focusing, or eye coordination abilities, all of which can lead to the development of visual symptoms when using a computer or digital screen device for a longer length of time. By the age of three, 68% of youngsters in England use computers on a regular basis, and 54% engage in online activities (2). Furthermore, other research found that adults in the United Kingdom spend between 4 and 45 min per day on screens (3), whereas adults in the United States spend almost two-thirds of their time on digital devices (5 h or more) (4).

According to recent US data, 37% of people aged 60 and overspend five or more hours per day on digital devices, and this age group likes to browse the internet on laptops and desktop computers, whereas younger folks prefer to do it on smartphones (4). Younger people are more likely to use social media and multitask, with 87 percent of those aged 20–29 indicating that they use two or more digital devices at the same time (4). The 2016 Digital Eye Strain research, which included answers from over 10,000 people in the US, found a 65 percent frequency of self-reported symptoms, with females being more impacted than males (69 vs. 60% prevalence) (4). Participants who used two or more devices at the same time were more likely to report DES than those who only used one device at a time, with prevalence rates of 75 and 53%, respectively. Various symptoms of DES arise after using mobile phones for more than 2 h daily (5), or digital devices after 2–4 h of exposure (6).

Sheppard and Wolffsohn (7) found that 27.5% of people have irritated or burning eyes, 31.5% have dry eyes, 30.6% have eye strain, 22.3% have headaches, 39.8% have tired eyes, 26.3% have sensitivity to bright lights, and 30.8% have eye discomfort. Eye health is negatively affected by online education and eye fatigue increased as a result of the COVID-19 pandemic process (8). Research was done in India to examine the prevalence of DES among computer users in the state of Bihar. The frequency of DES was discovered to be 69 percent. Around 30 percent of people utilized the computer for 4–6 h every day. Eyestrain and weariness were the most prevalent complaint in 59 (59%) people, followed by headache in 57 (57%) people, discomfort in the neck, shoulder, wrist, or back in 51 (51%) people, dry eyes in 37 (37%) people, and blurred vision in 35 (35%) people. CVS was mentioned by 11 people (11%). The most prevalent preventative intervention was taking pauses in between work, which was taken by 79 participants (79%). In the current study, 46 (46%) individuals took preventative breaks after 1 h and 25 (25%) after 20 min (9). According to research from Egypt, 86%of medical students who spent 3 h or more per day on the computer were suffering from one or more DES symptoms (10). Other symptoms were dry eyes, headache, blurred vision, eye strain, neck and shoulder pain, weariness, and eye redness. A study from Bulgaria shows similar results. Of those studied, 7.4% of students had ca onstant feeling and 25% often had feelings of eye soreness and irritation. Eye dryness, grittiness, and scratchiness was constantly experienced by 9.6% of the students and 19.1% felt it frequently (11). Study results from Israel and the USA reveal eye fatigue (60 and 48%), eye strain (58 and 31%), ocular discomfort (44 and 31%), headaches (43 and 26%), dry eyes (39 and 34%), and burning eyes (40 and 22%) (12). Computer-using and specialized ophthalmologists know more about symptoms and diagnostic signals than traditional ophthalmologists, yet they lack different treatment options (13). Recent results from Indian research found that the average age of children with DES was 13 ± 2.45 years. The average time spent on a digital device was 3.9 ± 1.9 h, up from 1.9 ± 1.1 h in the pre-COVID era (*P* = 0.0001). Smartphones were the most popular digital device among the participants (*n* = 134, or 61.7%). A total of 108 youngsters (49.8%) spent more than 2 h every day on online programs. The prevalence of DES was 50.23% in that group. There were 26.3% light cases, 12.9% moderate cases, and 11.1% severe cases. Itching and headache were the most often reported symptoms (*n* = 117, 53.9%). Age >14 years (*P* = 0.04), male gender (*P* = 0.0004), smartphone usage (*P* = 0.003), device use >5 h (*P* = 0.0007), and mobile games >1 h per day (*P* = 0.0001) were all found to be independent risk factors for DES in youngsters (14). Playing applications and games, as well as surfing the internet, are a common practice for our youngsters in the present digital world (8). Furthermore, most children lack the self-control necessary to set boundaries for themselves.

A study revealed that an 86% (*n* = 584) prevalence of DES was observed in those who had at least one symptom. As per the study, computer devices are used by participants mainly for learning and entertainment. One third of participants were continuously using digital screens for >2 h and one-fourth of participants were using the screen for >9 h; 20% used the screen in a dark room or dim light for >5 h. 66% had mild and 2.2% had severe symptoms. Headache was the common symptom found, followed by eye pain and neck/shoulder/joint pain. Females were found to be more prone to develop CVS. Headache, eye redness, burning, etc. were positively correlated with the duration of use (15). During the current pandemic, the creation of e-classes for such youngsters has placed an undue weight on their already strained eyes.

DES diagnosis and measurement: Both objective and subjective approaches have been used to assess DES. Objective evaluations of parameters such as critical flicker–fusion frequency blink rate and completeness, accommodative function, and pupil characteristics may be used to provide indices of visual fatigue. Subjective methods include a 10-item questionnaire produced by Hayes et al. (16) and utilized in various studies. It considers the symptoms of DES and scores each symptom separately. Another six-item Visual Fatigue Scale allows users to assess their difficulty in seeing, unusual feelings around the eyes, eyes feeling weary, feeling numb, having a headache, and feeling dizzy when gazing at the screen using a Likert scale (7). The Rasch-based Computer-Vision Symptom Scale is another tool that researchers may use to assess visual and ocular complaints in computer users. The self-administered Computer Vision Syndrome Questionnaire (CVS-Q) asks users to rate the frequency and severity of 16 symptoms they encounter when using a computer, resulting in a single symptom severity score (CVS score) of six or higher, which is considered diagnostic of the disease (17). The physiological underpinning of DES is used in objective evaluations. The exact process behind DES, however, remains unknown. In contemporary DES research, critical flicker–fusion frequency (CFF) and blinking characteristics have been utilized often to assess visual functions (7). Ergonomic practices, maintaining regular blinking, the use of adequate lighting, careful placement of the digital device, altering image characteristics (resolution, text size, contrast, and brightness), and taking breaks are all frequent non-pharmacological and pharmaceutical treatments. Artificial tears are one of the pharmacological management techniques.

According to Reddy et al. (18), only taking breaks from screens is insufficient for reducing DES symptoms, but concentrating on long-distance objects between breaks relates to a considerably better prognosis. The 20/20/20 method (looking at items over 20 feet away for 20 s after 20 min of visual display unit use) is very widespread advice in the literature (19). Furthermore, using antiglare displays in electronic devices to prevent eye strain is a common but less acknowledged ergonomic approach. The evidence for the antiglare screen's preventative advantages in DES is mixed. Ranasinghe et al. (17) and Shantakumari et al. (20) observed that individuals who used antiglare displays had fewer DES symptoms, but Reddy et al. (18) and Scullica et al. (21) reported that screen filters have no effect on DES symptoms. Some research has suggested that increasing ergonomic health literacy behaviors, as well as creating an ergonomic work environment, is a good way to avoid DES among screen users. It has been discovered that wearing computer glasses corrects refractive errors and reduces symptoms, whilst using precision spectrum filters reduces the symptoms of micro-fluctuation of accommodation. Anti-glare lenses are contentious, and there is no universal agreement on how to utilize them. Dry eye symptoms can be efficiently managed with artificial tears and omega-3 fatty acid consumption. On-screen prompts, audial prompts, or wink glass can all help users raise their effective blinking rate, which is known to be one of the most critical elements in preventing DES. In a qualitative European study of 368 children aged 9–16, subjects were queried on what they perceive as negative while using the internet and technology in general. To reduce the variety of eye problems, such as eye strain and eye irritation, the usage of eye glasses following prolonged internet use may help (22). A novel therapy modality called “*Warming Device”* might be a good alternative to the present therapeutic techniques for computer vision disorders (23, 24).

## Conclusion

A “Shadow Pandemic” is brewing because of DES. We are inadvertently driving a generation of youngsters toward a higher risk of DES due to the present trend of e-learning programs and its repercussions.

## Recommendations

A wide range of evidence is available to assess for the prevalence of DES among adult screen users, although comparable data for youngsters is scarce. Given the current COVID-19 pandemic and the growing burden of screen exposure of more than 12 h per day among youngsters, it is vital for policymakers in the education and health sectors to provide guidelines (e.g., limiting e-learning time for students to reduce screen time). Similar guidelines should be framed for the adults who are working from home with digital devices for a long time. Ophthalmologists can also be informed about the diagnosis and innovative therapy options for computer vision syndrome.

## Limitations

There are a few restrictions to this evaluation. It is a narrative review in which the evidence is retrieved and synthesized without using a systematic technique. We used a negative review technique due to the scarcity of research in this field, which is still a problem. There are also other limitations to this review. It is narrative review, although it emphasizes one of the most important public health issues in the post-pandemic era globally, which will likely drive future strategies aimed at avoiding DES in this relatively young population. The findings should be reproduced, and they should be compared to nations in Europe that have been subjected to protracted lockdowns, such as Italy, Austria, and Germany. As a result, more research, including both primary studies and evidence synthesis, is needed to inform decisions and practice.

## Author Contributions

All authors contributed equally.

## Conflict of Interest

The authors declare that the research was conducted in the absence of any commercial or financial relationships that could be construed as a potential conflict of interest.

## Publisher's Note

All claims expressed in this article are solely those of the authors and do not necessarily represent those of their affiliated organizations, or those of the publisher, the editors and the reviewers. Any product that may be evaluated in this article, or claim that may be made by its manufacturer, is not guaranteed or endorsed by the publisher.
